# Optimization of automated preparation of long oval-shaped root canals using supplementary instrumentation

**DOI:** 10.1590/0103-6440202305231

**Published:** 2023-05-15

**Authors:** Marco Antonio Castro-Perez, Cleber Keiti Nabeshima, Emmanuel. J. N. L. Silva, José Edgar Valdivia, Giulio Gavini, Manoel Eduardo de Lima Machado

**Affiliations:** 1 Department of Restorative Dentistry, School of Dentistry, University of São Paulo, São Paulo, Brazil; 2 Department of Endodontics, Grande Rio University, Rio de Janeiro, Brazil

**Keywords:** endodontics, microcomputed tomography, long oval-shaped root canal, root canal preparation

## Abstract

This study compared the preparation of long oval-shaped root canals using WaveOne Gold and XP-endo Shaper systems, both supplemented or not with manual instrumentation. Twenty-four long oval-shaped canals of mandibular incisors were distributed into two groups according to the instrumentation: WaveOne Gold Primary or XP-endo Shaper systems. All root canals were manually instrumented with a size 25 K-file after automated preparation. The specimens were scanned by using a micro-CT device (17.42 µm) before and after automated preparation and manual instrumentation. The increased surface of the root canal and the percentage of untouched areas were assessed. Both WaveOne Gold and XP-endo Shaper systems increased the root canal surface and had similar untouched areas (p>0.05). Supplementary instrumentation increased the surface of the root canal and decreased the untouched walls (p<0.05). WaveOne Gold and XP-endo Shaper systems provided a similar preparation of long oval-shaped canals and manual instrumentation improved their preparation.

## Resumo

Este estudo comparou o preparo de canais radiculares ovais longos usando os sistemas WaveOne Gold e XP-endo Shaper, ambos complementados ou não com instrumentação manual. Vinte e quatro canais longos e ovais de incisivos inferiores foram distribuídos em dois grupos de acordo com a instrumentação: sistemas WaveOne Gold Primary ou XP-endo Shaper. Todos os canais foram instrumentados manualmente com uma lima K tamanho 25 após preparo automatizado. Os espécimes foram escaneados usando um dispositivo de micro-CT (17,42 µm) antes e após o preparo automatizado e instrumentação manual. O aumento de superfície do canal radicular e a porcentagem de áreas intocadas foram avaliadas. Ambos os sistemas WaveOne Gold e XP-endo Shaper aumentaram a superfície do canal radicular e apresentaram áreas intocadas semelhantes (p>0,05). A instrumentação complementar aumentou a superfície do canal radicular e diminuiu o percentual de áreas intocadas (p <0,05). Os sistemas WaveOne Gold e XP-endo Shaper tiveram o preparo de canais ovais longos semelhantes, e a instrumentação manual melhorou o preparo.

## Introduction

Several automated systems have been proposed and their mechanical action is responsible for about 97% of the bacterial reduction within the root canal ^1^. However, anatomical complexity is a problem as the preparation of non-round root canals is more difficult ^2^ ^,^ ^3^ ^,^ ^4^ ^,^ ^5^. This difficulty could result in the failure of the endodontic treatment ^6^.

Among various automated systems, XP-endo Shaper (FKG Dentaire, La Chaux-de-Fonds, NE, Switzerland) is a continuous rotary single-file system made of a NiTi alloy called MaxWire, which changes from the martensitic to the austenitic phase when submitted to the temperature of 35°C. Its eccentric rotary motion is able to adapt to the morphology of the root canal having a shaping ability of up to size 30/.04 ^7^. WaveOne Gold (Dentsply Sirona, Maillefer, Ballaigues, VD, Switzerland) is a centric reciprocating single-file system made of a thermally treated NiTi alloy called Gold wire, which is available in four sizes: small (20/.07v), primary (25/.07v), medium (35/.06v), and large (45/.05v) ^1^. Previous studies showed WaveOne Gold prepared more walls of round root canals than XP-endo Shaper ^8^, but it resulted in high-unprepared canal areas of oval-shaped root canals ^9^. In general, both systems leave untouched areas within the root canal (5,8-14). Changing the kinematics of the automated systems was proposed to increase the overall prepared surface of the root canal ^15^, however, Carvalho et al. ^5^ found no difference in the XP-endo Shaper by using protocols with and without brushing motion to improve the preparation of the flattened root canals. Other authors proposed a supplementary instrumentation after automated preparation because it increased the area of touched walls within non-round root canals ^4^. This supplementary instrumentation by using circumferential movement could improve the preparation of the long oval-shaped root canal ^16^.

Given that both XP-endo Shaper and WaveOne Gold systems using manual supplementary instrumentation in long oval-shaped root canals have not been assessed yet, the aim of this study was to compare these systems in the preparation of long oval-shaped root canals and to evaluate the influence of manual supplementary instrumentation after automated preparation. The null hypotheses were that there is no difference between WaveOne Gold and XP-endo Shaper systems in the preparation of long oval-shaped root canals, and that manual supplementary instrumentation does not influence the automated preparation.

## Material and methods

Sample size calculation was based on a pilot study with five specimens using the mean and standard deviation of the percentage of untouched areas within the root canal before and after supplementary instrumentation. Wilcoxon-signed-rank test (matched pairs) was selected from the t-test family available on the G*Power 3.1 software (Henrick Heine-Universitat, Dusseldorf, NW, Germany). The effect size for this study was 1.92, with the alpha-type error of 0.05, a beta power of 0.95, and an allocation ratio (N2/N1) of 1. A total of 10 specimens (five per group) were indicated as the ideal size required for observing significant differences. Additional teeth were allocated to each group to compensate for any possible sample loss.

After approval by the local research ethics committee (protocol number 3.895.034), extracted human mandibular incisors from a collection were scanned by using a micro-CT (SkyScan 1176; Bruker-microCT, Kontich, AA, Belgium) device at isotropic voxel size of 34.84 μm, 87 Kv, 278 μA, rotation of 180° and rotation step of 0.7° with aluminum and copper filters to select straight single root canals. The images were reconstructed by using the NRecon software (version 1.16.1) (Bruker-microCT) with a beam hardening correction of 40%, smoothing of 4, ring artifact correction of 8, an attenuation coefficient ranging from 0 to 0.180, and then, analyzed with the CTAn software (version 1.16.4.1) (Bruker-microCT). The buccolingual and mesiodistal lengths of the cross-section of the root canal were measured at 5 mm from the root apex to determine the dimension of the long oval-shaped root canal, with a ratio between 2:1 and 4:1, according to Jou et al. ^16^.

Twenty-four mandibular incisors were selected and standardized in 18 mm through wear of the incisal surface. The root canals were accessed before being explored and the patency was confirmed with a size 10 K-file (Dentsply Sirona). Tooth smaller than 18 mm or with open apex were replaced.

The specimens were randomly distributed into two groups (n = 12), namely, groups WOG and XPS. Next, they were scanned again at an isotropic voxel size of 17.42 µm, 80 kVv, 310 µA, rotation of 180° and rotation step of 0.5 ° with aluminum and copper filters to acquire images before preparation. The total volume and area of the root canals were obtained with measurements made on microcomputed tomographic images by using the CTAn software. Standardization of the specimens between the groups was confirmed with a t-independent test (P > 0.05).

The roots were fixed in polyvinyl siloxane (Perfil Putty, Vigodent Coltène, Rio de Janeiro, RJ, Brazil) immersed in warmed water, and kept at 37°C during all instrumentation. The glide path was performed by using a size15 K-file along the working length of 17 mm and the specimens instrumented according to the group as follows:

Group WOG: root canals were prepared by using WaveOne Gold Primary files (25/.07v) and an electric motor (X-Smart Plus, Dentsply Sirona) operating in a reciprocating motion. The instrument was introduced into the root canal in the apical direction with a gentle inward “stroking” motion of short 2-3mm amplitude until reaching the working length.

Group XPS: root canals were prepared by using XP-endo Shaper file (30/.01) and electric motor (X-Smart Plus, Dentsply Sirona) operating in continuous rotation at 900 rpm and torque of 1 Ncm according to the manufacturer. The instrument was introduced into the root canal by using long gentle strokes along the working length, with 15 additional movements being made within it.

A single operator experienced instrumented the root canals, and each instrument was used once and then discarded.

Irrigation during preparation was performed with a total of 20 mL of 2.5% NaOCl solution (Fórmula & Ação, São Paulo, SP, Brazil) at 37°C (Anurb, São Paulo, SP, Brazil) by using a syringe and 30-G NaviTip needle (Ultradent, South Jordan, UT, USA) at 2 mm from the working length with in-and-out movements. Automated preparation was considered complete when a size 30/.04 gutta-percha cone (Dentsply Sirona) was inserted into the working length. If this gutta-percha cone did not reach the working length, automated movements were repeated.

The specimens were again scanned according to the pre-preparation parameters for the acquisition of images after automated preparation.

Next, the root canals were manually instrumented by using a size 25 K-file (Dentsply Sirona) until the working length with two circumferential movements against the buccal, mesial, lingual, and distal walls before traction in the coronary direction. The irrigation of the root canal in this step was carried out according to the automated preparation.

The specimens were again scanned according to the pre-preparation parameters for the acquisition of the images after additional instrumentation.

The images acquired after automated preparation and supplementary instrumentation were reconstructed by using the NRecon software. Next, they were superimposed by using the DataViewer software (Bruker MicrocT) and analyzed with the CTAn software.

The difference between the areas before and after both preparations determined the increase in the surface of the root canal. Quantitative analysis of untouched areas was carried out by overlapping cross-sections before and after both preparations. These areas were automatically calculated in mm^2^ and the resulting data were converted into percentages.

Normal data distribution was checked with Shapiro-Wilk test. The groups were compared by using independent-t test, whereas the paired-t test was used to assess the influence of manual instrumentation on the automated preparation. All comparisons were made regarding the root canal as a whole, as well as apical, middle, and coronal thirds. The level of significance in all analyses was 5%.

## Results

WaveOne Gold and XP-endo Shaper systems increased the root canal surface by 11.64% and 11.10%, respectively, and by 16.32% and 15.72% when supplementary instrumentation was performed. These systems had similar results after automated preparation and supplementary instrumentation (p>0.05), but the latter intervention increased the surface of the root canal (p<0.05).

The increase of the surface in the whole root canal and coronal, middle, and apical thirds can be seen in Table 1.

**Table 1:** Mean and standard deviation of the percent increase of surface in the whole root canal and coronal, middle, and apical thirds

	WaveOne Gold				XP-endo Shaper	
Thirds	Automated Preparation	Supplementary Instrumentation	p-value	Automated Preparation	Supplementary Instrumentation	p-value
Whole	11,64±6,67	16,32±8,10*	<0.0001	11,18±5,85	15,71±6,80*	<0.0001
Coronal	15,61±10,79	19,21±12,79*	0.0018	11,10±6,83	15,96±9,56*	0.0014
Middle	7,78±3,90	11,99±4,73*	0.0022	11,27±6,59	14,33±7,19*	0.0013
Apical	9,96±8,13	18,37±8,70*	<0.0001	12,72±9,31	20,41±10,15*	<0.0001

*Asterisks means the difference between columns in the same automated system. Paired-test-test (p<0.05)

WaveOne Gold system had 20.02% of the untouched area in the whole root canal after automated preparation, whereas the XP-endo Shaper system had 18.29%, and the supplementary instrumentation decreased the untouched walls to 9.43% and 10.37%, respectively (p<0.05). Therefore, WaveOne Gold and XP-endo Shaper systems produced similar results after automated preparation and supplementary instrumentation (p>0.05).

**Figure 1:**
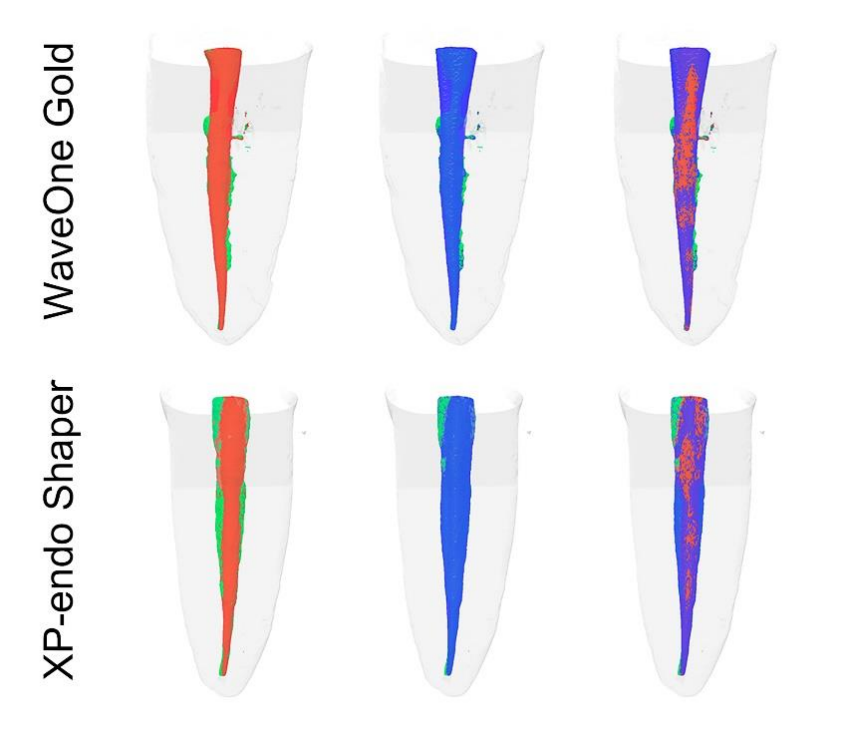
Lateral view of the three-dimensional reconstruction of the specimens in both groups (WOG and XPS). Touched areas by automated preparation (in red), touched areas by manual supplementary instrumentation (in blue), and untouched areas (in green).

The percentage of untouched areas in the whole root canal and coronal, middle, and apical thirds can be seen in Table 2, and Figure 1 shows the representative images of groups WOG and XPS after automated preparation and supplementary instrumentation.

**Table 2:** Mean and standard deviation of the percentage of untouched areas in the whole root canal and coronal, middle, and apical thirds

	WaveOne Gold			XP-endo Shaper		
Thirds	Automated Preparation	Supplementary Instrumentation	p-value	Automated Preparation	Supplementary Instrumentation	p-value
Whole	20,02±5,41	9,43±3,70*	<0.0001	18,29±8,43	10,37±7,04*	<0.0001
Coronal	13,79±5,19	5,41±2,08*	<0.0001	12,27±6,82	8,35±6,51*	0.0266
Middle	23,21±11,01	10,39±6,36*	0.0012	22,27±12,8	11,76±8,88*	0.0009
Apical	25,76±10,00	15,10±10,29*	<0.0001	21,34±10,30	12,87±8,48*	0.0015

*Asterisks means the difference between columns in the same automated system. Paired-test-test (p<0.05)

## Discussion

Different techniques and instruments have had difficulties in preparing root canals as untouched areas are always left after instrumentation ^2^ ^,^ ^4^ ^,^ ^5^ ^,^ ^8^ ^,^ ^9^ ^,^ ^10^ ^,^ ^11^ ^,^ ^12^ ^,^ ^13^ ^,^ ^14^ ^,^ ^15^. The present study assessed XP-endo Shaper and WaveOne Gold systems before and after additional manual instrumentation in the preparation of long oval-shaped root canals. The results showed that both systems had similar shaping abilities and the preparation was optimized after supplementary instrumentation. Thus, the first null hypothesis was accepted and the second one was rejected.

Analysis of root canal preparation was carried out by using a micro-CT device, which is the most currently used method as it is non-destructive and allows three-dimensional analysis with accurate measurements, in addition to maintaining images for future studies ^17^. Although long oval-shaped canal poses great difficulty in their preparation, the root canal of mandibular incisors and the distal root canal of mandibular molars have been mostly used in these studies ^2^ ^,^ ^10^ ^,^ ^13^ ^,^ ^14^ ^,^ ^15^ ^,^ ^18^. Wu et al. ^19^ found a higher incidence of long oval-shaped canals in mandibular incisors (56%) than in mandibular molars (30%) and for this reason, the mandibular incisor was the target of this study.

XP-endo Shaper system was chosen for its better adaptation to the root canal anatomy as proposed by the manufacturer ^7^, whereas the WaveOne Gold system was chosen because it is worldwide used and promotes a significant bacterial reduction within the root canal ^1^. The preparations using both systems increased the root canal surface, which corroborates other studies assessing several automated systems ^5^ ^,^ ^8^ ^,^ ^9^ ^,^ ^10^ ^,^ ^11^ ^,^ ^12^ ^,^ ^13^ ^,^ ^14^. Versiani et al. ^10^ and Marques et al. ^14^ found an increase of 10.8% and 11,3% respectively by using XP-endo Shaper files in long oval-shaped root canals, which are values close to ours, that is, 11.18%. On the other hand, Velozo et al. ^13^ reported an increase of 27.74%, which could be explained by an additional preparation for 45 seconds. However, this hypothesis needs further studies for confirmation since the cross-section root canal has led to different results when the working time of the XP-endo Shaper is increased ^5^ ^,^ ^11^, and the long oval-shaped root canals have not yet been assessed in this variation. The similarity between WaveOne Gold and XP-endo Shaper systems corroborates the study by Thomas et al. ^12^, who found no difference between WaveOne Gold and X3D-Shaper systems, in which the latter has characteristics similar to those of XP-endo Shaper. The manual supplementary instrumentation increased the root canal surface after automated preparation, which is in agreement with previous studies ^4^. These results can be explained by trials demonstrating that the surface of the root canal increases depending on the wear of the dentin ^20^ ^,^ ^21^.

The systems showed no difference in the untouched areas (20.02% in WOG versus 18.29% in XPS). Although Versiani et al. ^10^ and Marques et al. ^14^ also reported no difference between XP-endo Shaper and other continuous rotary systems used in long oval-shaped root canals, they found 9.42% and 9,57% of untouched areas in the whole root canal. This discrepancy between values may be due to the anatomical variation of the specimens. Versiani et al. ^10^ used the classification proposed by Wu et al. ^19^, which includes oval long and flat-shaped canals in the same category, and Marques et al. ^14^ used the distal root canal of mandibular molars. According to Wu et al. ^19^, these root canals have a larger diameter than root canals of mandibular incisors. With regard to the WaveOne Gold system, Thomas et al. ^12^ found no difference between it and other continuous rotary systems, although 50.9% of the areas remained untouched. This value can also be related to the anatomical condition because the authors used oval-shaped root canals of premolars. The XP-endo Shaper system is indicated for root canals with complex anatomy, that is, those with oval, oval long, and flat-shaped root canals. This study and others in the literature demonstrated that the increased extent of the instrument in the austenitic phase did not improve the preparation of the root canal ^10^ ^,^ ^13^ ^,^ ^14^. However, the manual supplementary instrumentation decreased the untouched areas from 20.02% to 9.43% and from 18.29% to 10.37%, respectively, in the groups WOG and XPS. However, it is important to highlight that K-file was chosen because it has greater rigidity, which facilitates the access to recessed areas ^22^, the size 25/.02 was used to freely reach the cross-section ends of the root canal avoiding excessive wear of the proximal walls ^18^. These characteristics are important as these root canals have less dentin due to the previous automated instrumentation. Thus, our results confirm the contact between the manual instrument and untouched recesses left after automated preparation, and they are in agreement with studies assessing supplementary instrumentation after automated preparation ^4^ ^,^ ^15^. On the other hand, untouched areas were still present even after supplementary instrumentation ^4^ ^,^ ^15^. Studies on oval-shaped root canals showed that these untouched areas are located at the buccal and lingual ends ^23^ ^,^ ^24^, which was also observed in the present study on long oval-shaped root canals. This region has a higher prevalence of secondary root canals in mandibular incisors ^25^, which can be a source of substrates for the remaining bacteria ^3^.

Although manual supplementary instrumentation after automated preparation optimized the preparation of long oval-shaped root canals, it did not solve the problem of untouched areas. Therefore, more techniques aimed to act on these areas should be explored and further studies focused on the cleaning and disinfection of long oval-shaped root canals by means of manual supplementary instrumentation should be encouraged.

In view of the applied methodology, it can be concluded that both WaveOne Gold and XP-endo Shaper systems provide a similar preparation of long oval-shaped root canals and that manual supplementary instrumentation improves the automated preparation of these root canals.
